# Optical Coherence Tomography of Spontaneous Basilar Artery Dissection in a Patient With Acute Ischemic Stroke

**DOI:** 10.3389/fneur.2018.00858

**Published:** 2018-10-16

**Authors:** Peng Gao, Liqiang Gui, Bin Yang, Timo Krings, Liqun Jiao

**Affiliations:** ^1^Department of Neurosurgery, Xuanwu Hospital, Capital Medical University, Beijing, China; ^2^Department of Interventional Neuroradiology, Xuanwu Hospital, Capital Medical University, Beijing, China; ^3^Department of Neurosurgery, Langfang Changzheng Hospital, Langfang, China; ^4^Division of Neuroradiology, Toronto Western Hospital, Toronto, ON, Canada

**Keywords:** spontaneous intracranial arterial dissection, intravascular optical coherence tomography, acute ischemic stroke, imaging diagnosis, feasibility, safety

## Abstract

The diagnosis of intracranial arterial dissection (IAD) may be challenging and multimodal imaging techniques are often needed to confirm the diagnosis. Previous studies have based their criteria for diagnosis of IAD on conventional angiography, computed tomography, or magnetic resonance imaging. We report a case with acute ischemic stroke due to spontaneous basilar artery dissection in which intravascular optical coherence tomography (OCT) was used to show features of IAD. A 59-years-old woman presented with symptoms of acute ischemic stroke. Thrombosis related to basilar artery (BA) stenosis was assumed on conventional angiography; however, no clot was retrieved after mechanical thrombectomy (MT) and a restored BA caliber was observed after a rescue recanalization with the detachment of a self-expanding stent was performed. Spontaneous IAD was suspected; however, angiographic findings were ambiguous for confirming IAD. The patient remained symptom-free until 18-months follow-up. At this point, angiography showed restenosis at the proximal tapered length of the stent. *In vivo* OCT was performed to assess the pathological changes of the restenosis and confirm the diagnosis of IAD.OCT revealed BA dissection with the presence of remnant transverse flap, double lumen and mural hematoma. Imaging at multiple levels identified intimal disruption that originated in the right vertebral artery and extended distally to the BA. The use of intravascular imaging with OCT enabled the accurate diagnosis of IAD. Care should be taken as the procedure may add additional risks to the patient. Future studies are needed to validate the safety of OCT in IAD.

## Background

Spontaneous intracranial artery dissection (IAD) is uncommon, with its incidence estimated to be lower than that of cervical artery dissection (2.6–3.0/100,000/year) among patients of European ethnic origin (1, 2). In Italy, the prevalence of IAD was 23/4400 (0.5%) among patients with a diagnosis of cerebral ischemia (3). Another study from France demonstrated spontaneous IAD to account for 3.3% (13/391) of patients with acute ischemic stroke due to large vessel occlusion (LVO) (4). However, in Asia, IAD is not such a rare diagnosis with its reported prevalence of up to 20.5% (192/937) among patients with intracranial atherosclerotic narrowing on TOF-MRA (5). In Korea and Taiwan, IAD accounted for up to 67–78% of all cervicocephalic artery dissections (6, 7). Therefore, IAD may be an important cause of acute ischemic stroke and should be considered in young patients with acute intracranial LVO.

The diagnosis of IAD may be challenging due to the small size of intracranial arteries and non-specific radiological signs. Multimodal imaging techniques are often needed to confirm the diagnosis of IAD (8). In most cases, IAD is defined based on angiographic criteria rather than magnetic resonance imaging given its clinical presentation as an acute stroke (4, 8). The prevalence of the pathognomonic finding of IAD—the mural hematoma may be underdiagnosed by luminal examination alone and many Asian patients with IAD may be misclassified as having intracranial atherosclerotic disease (5). Intra-arterial Optical Coherence Tomography (OCT) is a novel catheter-based imaging technique that uses coherent light to capture micrometer-resolution with two—and three-dimensional images of the vessel wall. The sharp delineation of the luminal borders on OCT enables assessment of the vessel dissection, tissue prolapse, and stent-vessel interactions (9). Although it was initially approved for imaging of coronary arteries, OCT has been used to identify extracranial traumatic aneurysms (10), assess carotid arteries (11), and evaluate intracranial atherosclerotic disease (12). Nevertheless, *in vivo* imaging on IAD pathology has not been well described in the medical literature. In the present case, OCT was used to assess the intravascular changes of the intracranial arterial wall in a patient with basilar artery (BA) dissection. The utility and safety concern of OCT as an imaging modality in the diagnosis of IAD are discussed.

## Case presentation

### History and findings

A 59-years-old woman presented with sudden onset of lethargy, slurred speech, and left extremity weakness since 5 h. Neurological examination indicated right gaze preference, dysarthria, and decreased muscle strength on the left side (grade II). The patient had a NIHSS score of 8. Previously, the patient presented with paroxysmal dizziness for 1 year and had no history of brain trauma. No intravenous tissue plasminogen activator (tPA) was given since symptom onset was 5 h after presentation to the emergency room.

### Angiography and recanalization approaches

**Figure 1:**
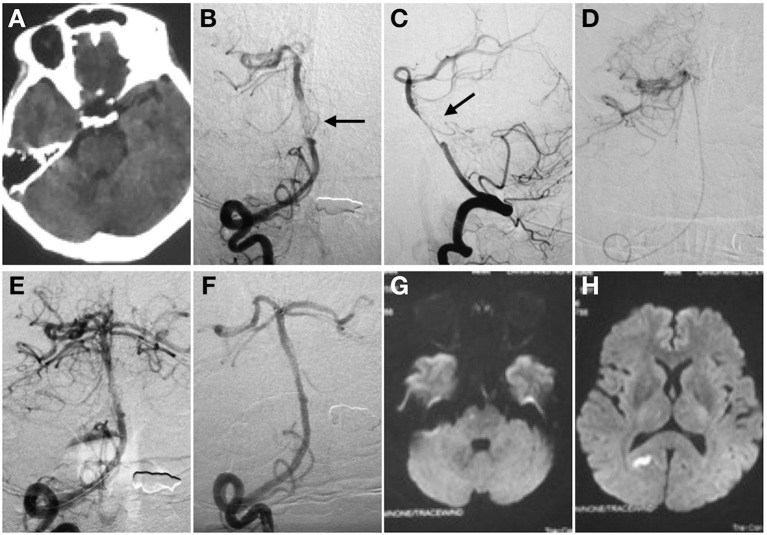
Spontaneous basilar artery (BA) dissection causing acute ischemic stroke. Non-contrast CT **(A)**. Anterior **(B)**, and lateral **(C)** projection of angiography before stent deployment. There is a “non-occlusive thrombus” in the mid-BA with regular residual lumen **(C)**. Intracranial atherosclerotic stenosis was then suspected (black arrow). The super -selective angiography control was performed to confirm the tip of the microcatheter in the real lumen **(D)**. Angiography after the first mechanical thrombectomy maneuver **(E)**. No clot was found. Angiography immediately after stent detachment showed the stent restored the BA caliber **(F)**. The distal marker of the stent lined up with the BA tip whereas the proximal marker was located within the right vertebral artery. MRI in transverse DWI section showed acute infarctions in right pons **(G)** and occipital lobe **(H)** after procedure.

The patient was admitted and transferred to the catheter room 5.5 h after the onset of symptoms. Digital subtraction angiography (DSA) demonstrated a filling defect caused by a long segment severe stenosis in the BA, which was first assumed to be intraluminal clot related to BA stenosis. After a 6Fr guiding catheter (Envoy, Cordis) was placed into the right vertebral artery (VA), a microcatheter (REBAR-21, Covidien) co-axially assembled with a 0.014-inch Synchro Standard microwire (Stryker, Neurovascular) was used to traverse through the lesion. A self-expanding stent retriever (SOLITAIRE AB 6–30 mm, Covidien) was deployed across the lesion. Mechanical thrombectomy (MT) was performed; however, no clot was found. Repeat DSA showed even worse antegrade flow. It was decided to deploy the stent retriever which lead to restored caliber of the BA. IAD rather than ICAS was suspected. Nevertheless, conventional DSA failed to confirm the diagnosis of IAD. After the procedure, the patient regained consciousness and speech without gaze preference. The muscle strength on the left side recovered to grade III. Intravenous platelet glycoprotein IIb/IIIa receptor inhibitors (Tirofiban, Yuanda Pharmaceuticals, Wuhan, China) was maintained (5 ml/h) for 18 h after the procedure. Double anti-platelet regimen (aspirin 100 mg plus clopidogrel 75 mg per day) was given for 3 months (aspirin 100 mg alone thereafter). Post-operative Diffusion-weighted imaging (DWI) showed acute infarctions in the right pons and occipital lobe (Figure 1). The patient had a NIHSS score of 2 at discharge and 0 at 3-months follow-up, respectively. The modified Ranking Score at 3 months was 1.

### Follow-up angiography

**Figure 2:**
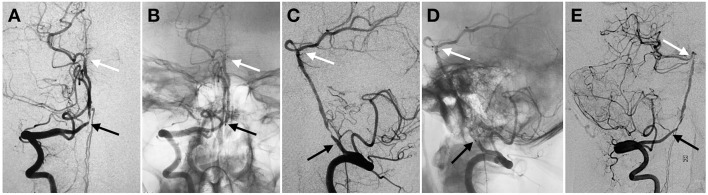
Proximal in-stent restenosis on 18-months follow-up angiography. Anterior **(A,B)** and lateral **(C,D)** view of subtracted **(A,C)** and un-subtracted angiography **(B,D)** in right VA demonstrated proximal stenosis within the tapered length of the stent construct. Oblique view of the angiography **(E)**. Black and white arrow indicated proximal and distal markers of the stent, respectively.

The patient had no recurrent symptoms until 18-months follow-up. She was transferred to our institute due to paroxysmal dizziness and blurred vision for the past month. In-sent restenosis was confirmed on follow-up angiography (85% based on WASID criteria) (13). The restenosis was located within the proximal tapered area of the SOLITAIRE stent (Figure 2).

### Optical coherence tomography (OCT) procedure and interpretation

The need for further intervention of this restenosis was uncertain based on DSA alone. Therefore, OCT was performed in order to assess the underlying cause of restenosis and confirm the diagnosis of IAD. The patient has signed informed consent regarding the use of OCT which was approved by the local Institutional Review Board. The intravascular frequency-domain OCT system (ILUMIEN OPTIS, OCT Intravascular Imaging System; St. Jude Medical) was used. After conventional angiography, the patient was placed under general anesthesia. A bolus of 5,000 units of intravenous heparin was administrated. With a 6Fr intermediate catheter (NAVIEN 115 cm long, Covidien) placed in the right VA, a 0.014 inch 300 cm long microwire (PILOT 150, Abbott) co-axially assembled with a microcatheter (ECHELON-10, Covidien) was carefully advanced through the proximal stent marker, the area of restenosis, and placed in the right posterior cerebral artery (PCA). After that, the microcatheter was exchanged for a 2.7Fr OCT imaging catheter (Dragonfly Duo; LightLab Imaging, Inc., St. Jude Medical).The short “monorail” design of the Dragonfly catheter did not permit its proximal marker to enter the PCA despite many attempts. After the catheter was advanced as far as in the mid-BA, control angiography demonstrated the opacification of the BA dissection.

**Figure 3:**
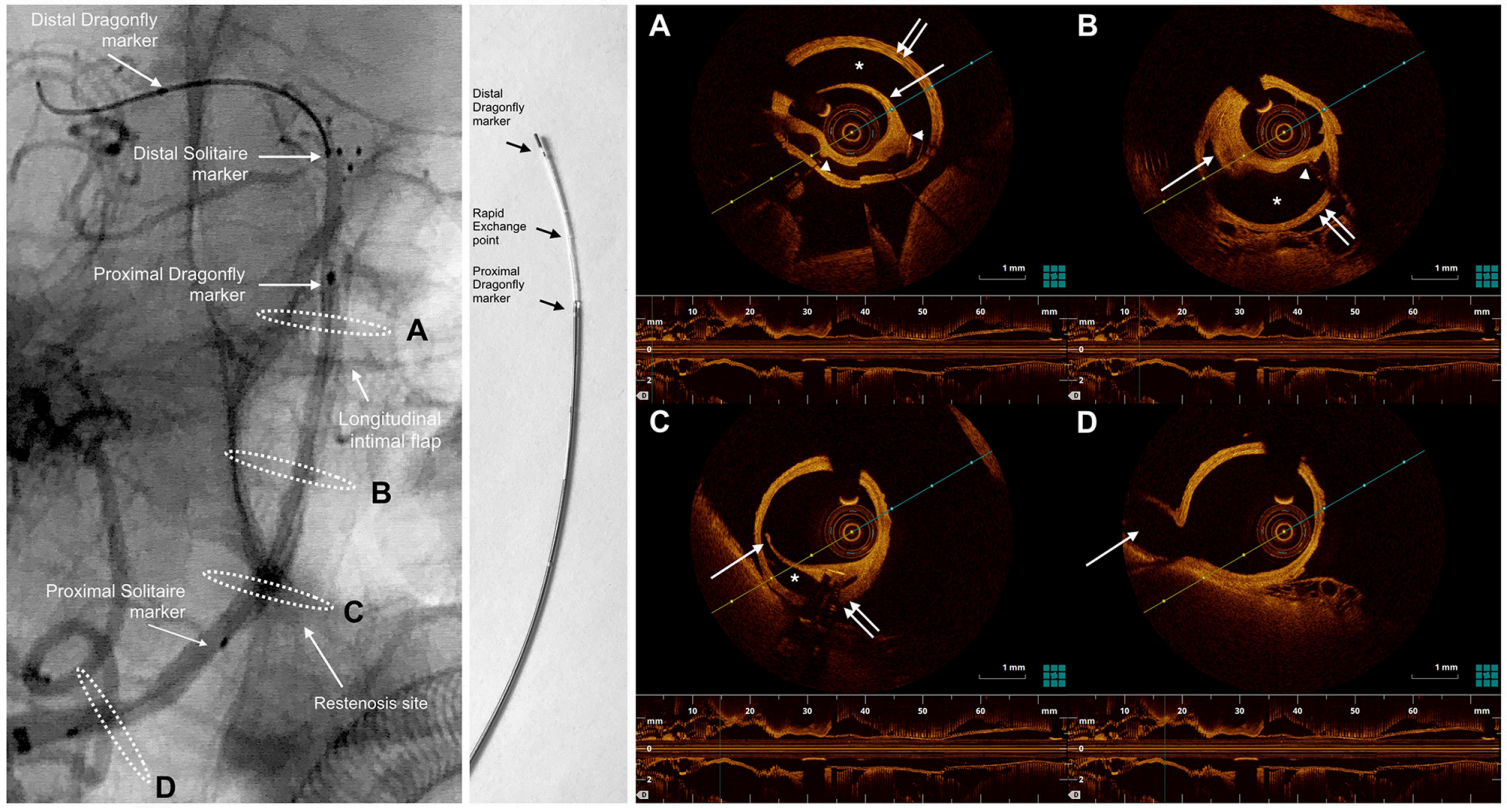
oblique view from an un-subtracted angiography of the right vertebral artery shows right VA dissection extending to BA. The markers of the SOLITAIRE stent and the Dragonfly Duo OCT imaging catheter are noted in the left panel. White dashed ellipses on the left panel correspond to the cross-sections of the OCT acquisition (right). The Dragonfly Duo imaging catheter is illustrated in the middle. **(A)**, the OCT image demonstrated a severe dissection (*), between the intima (single white arrow) and media (double white arrow). Poor strut-wall apposition was noted (struts indicated by the white arrowheads). **(B)**, 10 mm proximal to the cross-section A, there was a mural hematoma (single white arrow) within the dissection. The intima was complete without disruption. The false lumen (*) between the intima and media (double white arrow) caused the moderate stenosis of the BA. **(C)**, at the level of restenosis in right VA within the tapered area of the SOLITAIRE stent, there is a disrupted transverse flap (single white arrow) and mural hematoma, suggesting the dissection originated in right VA and extended distally to BA. **(D)**, at the level of posterior inferior cerebral artery (PICA) origin, no dissection is visible on DSA or OCT. Single white arrow indicated PICA. The presence of remnant transverse flap, double lumen and mural hematoma were signs of intracranial BA dissection on OCT.

Imaging at multiple levels was performed along the BA with an automatic pullback speed (36 mm/s) during blood clearance by the injection of contrast medium. The OCT data were analyzed by the ILUMIEN OPTIS Imaging System. OCT imaging demonstrated visualization of a dissection and poor stent strut wall apposition (Figure 3). The intimal disruption was limited to the VA and the false lumen extended into the BA. There were no clot formation or tissue prolapse within the stent. After the OCT imaging catheter was withdrawn, control angiography demonstrated rapid antegrade flow and improved lumen at the site of the previously demonstrated restenosis. No progressive stenosis or occlusion was noted after 10 min observation and no additional intervention was needed. The patient was given intravenous Tirofiban for 24 h after procedure. She had no symptoms and was discharged without neurological deficits 3 days after the procedure.

## Discussion

*In vivo* intravascular imaging of the vessel wall includes intravascular ultrasound (IVUS) and OCT. IVUS is the first intravascular imaging modality, which involves the advancement of a miniaturized ultrasound probe to the arteries. The probe is attached to the distal end of the catheter and is able to emit ultrasound signals perpendicular to its axis allowing visualization of the arteries (14). OCT is a recently-developed imaging technique that utilizes a light source with a near-infrared spectrum to produce cross-sectional images of arteries by measuring the echo time delay and the intensity of backscattered light (15). In this respect, OCT is somehow analogous to ultrasound imaging; however, OCT is superior to IVUS due to its high spatial resolution (4–10 μm vs. 70–100 μm) to distinguish the intima, media and adventitia of the arterial wall (16). Because of its easy passage of imaging catheter through the carotid arteries, OCT has been more frequently used in the extracranial carotid artery diseases than that in the intracranial vasculature. However, the intracranial vasculature is better accessed with full extent of OCT because the circumferential tissue visualization of OCT is limited to 5 mm from the catheter. Prior studies have noted the importance of OCT. It has been used to image the vessel wall of intracranial aneurysms, inflammation and atherosclerosis. In 2005, OCT demonstrated cerebral aneurysm healing following coil embolization in a canine model (17). After that, the use of OCT to assess mal-apposition of flow diverters has been shown to be predictive of the 30-days healing rate in a rabbit model of aneurysms (18). Nevertheless, the application for intracranial lesions in humans was not reported until 2014 (12). The study by Given et al showed OCT has potential applications in the evaluation of ICAS after Wingspan stent placement (12). Since 2014, there has been no other reported use of OCT in other intracranial lesions.

In our case, OCT was used to evaluate one patient who presented with spontaneous IAD in BA. The diagnosis of IAD other than ICAS clot was not confirmed during the initial mechanical thrombectomy. In our center, the use of OCT as an adjunct to DSA was performed. The intravascular changes of IAD were visualized on OCT for the first time. Consistent with the current pathognomonic criteria, OCT revealed signs of mural hematoma, intimal flap, and double lumen (8). The intimal disruption was observed in the proximal segment of the stent, which was not visible on DSA. Therefore, the findings of OCT may not only help to diagnose IAD, but also to provide better understanding of IAD and eliminate differential diagnosis of intracranial narrowing.

The potential advantages of OCT include fast acquisition time that allows for repeated scans, and high spatial resolution and depth-resolved analysis that enables us to see the details of disease more accurately. Studies from coronary intervention have demonstrated that OCT not only helps to diagnose coronary dissection, but can also guide endovascular treatment (19, 20). Stenting, as a salvage strategy, has been used to rescue patients with coronary dissection (21). Nevertheless, angiography-guided stenting of coronary dissection is often difficulty and carries additional risks. OCT-guided stenting is safe and effective in that it ensures the guidewire in the true lumen, allows for the accurate caliber of the stent, and enables the stent to cover the dissection on all its length. Nevertheless, as for patients with acute stroke due to IAD, the optimal treatment remains uncertain due to the limited case reports. In the study by Labeyrie et al. stenting of IAD was used as first-line approach to rescue patients with acute stroke due to intracranial large vessel occlusion. However, without the guidance of OCT, stenting of a circulating false lumen failed to recanalize the artery in 2 (25%) out of 8 patients (4). Therefore, one can expect that OCT-guided stenting may be secure and reliable to manage IAD because it may circumvent risk of stenting in a false lumen and achieve covering of dissection with full length.

It has to be kept in mind, though, that OCT is an invasive technique and should be used with caution. The image quality of OCT may also be hampered by the presence of red thrombus and hypercoagulability. In order to obtain reliable images, OCT procedures require injection of contrast media to wash out blood from the vessel lumen because the near-infrared light signals are attenuated by the presence of red blood cells. This procedure may aggravate the false lumen with high pressure contrast injection. Therefore, OCT should be only used in complex cases with ambiguous diagnosis that cannot be managed with conventional examinations. Once diagnosed with IAD, OCT may be helpful in select cases to guide endovascular treatment with stenting.

High-resolution MRI (HR-MRI), a non-invasive MR based technique, is a promising tool to directly evaluate the intracranial vascular wall in various conditions (22). Pathognomonic radiological findings of IAD consist of mural intimal flap, hematoma, and double lumen (8). In the study by Han et al. HR-MRI corroborated the final diagnosis in 94% of patients with intracranial vertebrobasilar artery dissection, in which intimal flaps were identified in 91.4% of patients on contrast-enhanced (CE) T1-Weighted (T1WI) HR-MRI (23). On T1WI MRI, mural hematomas are spontaneously hyperintense 48–72 h after onset, which are then best detected on T1WI and CE-T1WI (54.3%) with black-blood effect by using high resolution 3-Tesla imaging (23, 24). In our case, HR-MRI may be an alternative to OCT because numerous studies have shown its value in differentiating IAD from ICAS, moyamoya disease, vasculitis and reversible cerebral vasoconstriction syndrome (25). However, current HR-MRI has several limitations. Validation of HR-MRI imaging criteria of IAD with histological analysis appears difficult in view of the limited availability of histological specimens. Furthermore, HR-MRI can be time-consuming when we need high-resolution and evaluate small tortuous intracranial arteries. Sometimes, poor quality of imaging may be obtained due to patient motion and discomfort (26). At last, despite the successes and further technical developments, HR-MRI is limited by the lower spatial resolution (around 300 μm) when compared with OCT (10 μm) (16).

A recent study suggested the use of self-expanding stents to treat IAD as a first-line approach to recanalize IAD (4). In our case, there were two aspects that may explain the decreased efficacy of permanent stenting in the treatment of IAD. First, follow-up OCT demonstrated the intimal disruption within the tapered area of the stent. That indicated the dissection started from the right VA and extended into the BA. The initial deployment of one self-expandable stent (SOLITAIRE 6–30 mm) covered the proximal dissection only with the tapered end instead of the non-tapered portion of the stent. Therefore, the dissection was not completely healed. Ideally, proximal placement of an additional stent may have covered the intimal flap and prevented restenosis. Second, the SOLITAIRE stent has less radial force when compared to other intracranial stents. The proximal tapered 10 mm area (total length minus usable length) has even less radial force, which may delay the healing of IAD and cause subsequent restenosis despite administration of anti-platelet regimen. We observed, even after 18-months follow-up, that the false lumen persisted, suggesting poor interaction between the stent and the vessel wall. Moreover, OCT indicated a mural hematoma existed between the layers of intima and media, which has not dissolved after 18-months follow-up.

The visualization of IAD on OCT may be counterbalanced by the inherent risks during OCT procedures. The major challenges include vessel tortuosity and the monorail design of the imaging catheter. In our case, the combined use of microwire and microcatheter enabled the microwire to advance smoothly and avoided the risk of entering the circulating false lumen. In addition, an intermediate catheter with a stiff microwire (Pilot 150, Abbott) may achieve better support and facilitate advancing the imaging catheter through tortuous anatomy. However, the short monorail design of the rapid exchange of the Dragonfly catheter does not allow its distal marker going further, and left its proximal marker within the BA. *Given et al* described a similar condition, in which the proximal marker of the OCT catheter failed to advance into the PCA resulting in incomplete imaging of the BA (12). In addition, during our procedure, repeated attempts to navigate the catheter through the lesion together with high pressure contrast injections, probably caused the recurrence of the intimal flap and aggravation of the dissection on OCT. In view of the limitations of the monorail design of the current OCT catheter, it is empirically hypothesized OCT is feasible in patients with extracranial dissections and selective intracranial dissection, but not those with IAD beyond the siphon segment of internal carotid artery (ICA) or distally-located IAD. Although Lopes et al. devised a method to overcome the monorail design and inserted this catheter in the middle and posterior cerebral arteries of a fresh frozen cadaver, it has not been tested *in vivo* (27). Future observational series are needed to validate the safety of OCT in IAD.

OCT was initially designed for the evaluation of the coronary vasculature. The indications of OCT expanding from coronary to other vascular territories remain unknown. Currently, the Dragonfly Duo catheter may not be well compatible with intracranial vasculature. In the future, new, more flexible catheters, shorter scan distances, and smaller diameter may overcome the intracranial tortuosity, ease the concern of the peri-procedural complications and result in better technical results.

## Conclusion

OCT may be helpful to diagnose IAD where angiography is ambiguous. Understanding the role and careful use of OCT is expected to improve the diagnosis of IAD when clinically indicated.

## Ethics statement

This study was carried out in accordance with the recommendations of ethics committee of Xuanwu Hospital, Capital Medical University. The protocol was approved by the ethics committee of Xuanwu Hospital, Capital Medical University. All subjects gave written informed consent in accordance with the Declaration of Helsinki.

## Author contributions

PG drafted the initial manuscript. LJ and TK critically reviewed and revised the manuscript. LG performed the first mechanical thrombectomy for this patient. BY finished the OCT scan. All the authors reviewed and approved the final version of the manuscript.

### Conflict of interest statement

The authors declare that the research was conducted in the absence of any commercial or financial relationships that could be construed as a potential conflict of interest. The reviewer SW and handling editor declared their shared affiliation at the time of the review.
